# Causes and Consequences of Spatial Within-Host Viral Spread

**DOI:** 10.3390/v10110627

**Published:** 2018-11-13

**Authors:** Molly E. Gallagher, Christopher B. Brooke, Ruian Ke, Katia Koelle

**Affiliations:** 1 Department of Biology, Emory University, Atlanta, GA 30322, USA; mgallagher@emory.edu; 2 Department of Microbiology, University of Illinois at Urbana-Champaign, Champaign, IL 61801, USA; cbrooke@illinois.edu; 3 Carl R. Woese Institute for Genomic Biology, University of Illinois at Urbana-Champaign, Champaign, IL 61801, USA; 4 T-6, Theoretical Biology and Biophysics, Los Alamos National Laboratory, Los Alamos, NM 87545, USA; rke@lanl.org

**Keywords:** Influenza virus, within-host viral dynamics, spatial spread, within-host evolution

## Abstract

The spread of viral pathogens both between and within hosts is inherently a spatial process. While the spatial aspects of viral spread at the epidemiological level have been increasingly well characterized, the spatial aspects of viral spread within infected hosts are still understudied. Here, with a focus on influenza A viruses (IAVs), we first review experimental studies that have shed light on the mechanisms and spatial dynamics of viral spread within hosts. These studies provide strong empirical evidence for highly localized IAV spread within hosts. Since mathematical and computational within-host models have been increasingly used to gain a quantitative understanding of observed viral dynamic patterns, we then review the (relatively few) computational modeling studies that have shed light on possible factors that structure the dynamics of spatial within-host IAV spread. These factors include the dispersal distance of virions, the localization of the immune response, and heterogeneity in host cell phenotypes across the respiratory tract. While informative, we find in these studies a striking absence of theoretical expectations of how spatial dynamics may impact the dynamics of viral populations. To mitigate this, we turn to the extensive ecological and evolutionary literature on range expansions to provide informed theoretical expectations. We find that factors such as the type of density dependence, the frequency of long-distance dispersal, specific life history characteristics, and the extent of spatial heterogeneity are critical factors affecting the speed of population spread and the genetic composition of spatially expanding populations. For each factor that we identified in the theoretical literature, we draw parallels to its analog in viral populations. We end by discussing current knowledge gaps related to the spatial component of within-host IAV spread and the potential for within-host spatial considerations to inform the development of disease control strategies.

## 1. Introduction

More often than not, viral populations are spatially structured. At the between-host level, this spatial structure is evident for endemic pathogens from observed patterns of genetic differences across space, such as those observed for measles virus at large geographic scales [1] and dengue virus even at intracity scales [2]. In the case of epidemic pathogens, both surveillance data and viral genetic data often point to the occurrence of spatial spread, for example, in seasonal epidemics of influenza viruses in the U.S. [3,4]. In recent years, the processes driving these spatial dynamics have been increasingly well characterized and include mobility patterns [3,5,6] and activity patterns of hosts and vectors [7,8,9], among other factors. Characterizing these spatial dynamics and understanding the factors driving them are important for anticipating local timing of disease incidence and for guiding more informed control strategies.

At the within-host level, many viral populations also exhibit spatial structure. For chronic viral infections, such as cytomegalovirus and SIV/HIV, evidence for this structure comes from genetic compartmentalization [10,11,12,13,14]. In acute or slowly progressing chronic infections, spatial spread has been documented through spatially explicit ‘surveys’ of viral populations, for example, for influenza A viruses [15] and hepatitis C virus [16,17]. The specific processes driving these spatial dynamics have also been increasingly well characterized at the within-host level through empirical studies focused on elucidating factors that influence viral dissemination and cell/tissue tropism [18,19,20]. In contrast to the extensive experimental evidence demonstrating spatial spread as a major route of viral dissemination, only a few mathematical/computational modeling efforts have considered spatial viral spread [21,22,23]. The existing body of work indicates that spatial models give rise to dynamics that are distinct from models that assume no spatial structure. Considering spatial spread is particularly important for anticipating the timing of infection of specific tissues and in evaluating the efficacy of different classes of drug candidates [24,25]. Ignoring spatial viral spread can further lead to incorrect or biased estimates of parameter values from data [26].

Although these studies pointed out the importance of considering spatial structure in viral spread, there is a lack in theoretical understanding of the causes and consequences of spatial spread more generally. For example, little is known about how spatial viral spread impacts patterns of viral coinfection and cellular multiplicity of infection (MOI), and what the consequences of this are for viral population dynamics, the extent of viral recombination and reassortment within hosts, and the rate of within-host viral adaptation [27].

From an ecological perspective, populations are regulated by what are known as bottom-up and top-down processes [28]. Bottom-up processes determine the extent of resources available to a population, while top-down processes primarily determine the death rates of individuals in a population. One can also adopt this perspective to examine the ecology of within-host viral infections. For example, viral populations are subject to bottom-up processes, such as the availability of susceptible target cells, and top-down processes, such as viral clearance by immune cells. These top-down processes can be thought of as analogous to predator-prey relationships, where the immune system ‘preys’ upon viruses and infected cells. For all populations, including viruses, these bottom-up and top-down processes occur at characteristic spatial scales that determine the spatial dynamics of organismal spread and further impact their evolutionary dynamics.

Here, we first review patterns and mechanisms of within-host viral spread from this bottom-up/top-down perspective. We then turn to the computational modeling literature to review insights gained from modeling studies as to how bottom-up and top-down processes, acting at characteristic spatial scales, can drive patterns of within-host viral spread. We limit our reviews of the empirical and modeling studies to human influenza A viruses (IAVs) as representative and well-studied acute infections of the respiratory tract.

For these viruses, we surprisingly find only a very limited number of computational studies that explicitly consider the causes and consequences of spatial within-host spread. This is worrisome, given that existing studies indicate, as mentioned above, that spatial models can yield qualitatively different viral dynamics from ones that assume no spatial structure. We thus then turn to the extensive theoretical ecology and evolution literature to shed light on what characteristics of viral populations are likely to impact patterns of spatial viral spread and the evolutionary consequences of this spread. While much of our review focuses on influenza viruses, these theoretical insights should be applicable to other viral systems undergoing spatial within-host spread.

## 2. Experimental Studies Indicate That Within-Host IAV Spread Is a Strongly Spatially Structured Process

The within-host spatial dynamics of influenza virus infection have been increasingly well characterized over the last decade. Early work relied on immunohistochemistry and in situ hybridization approaches to determine the extent of spatial heterogeneity in viral presence/absence across infected host tissues. For example, by examining lung tissue blocks from several human patients who had fatal influenza infections, Guarner and colleagues found evidence for focal influenza infection in the epithelium of large bronchi in a subset of patients [29]. Interestingly, they found that viral antigen was only found within a fraction of the lung tissue blocks they examined, thus providing one of the first lines of evidence that influenza infections have a spatial dimension. While this study may have been biased based on the exclusive focus on fatal influenza infections, bronchoscopy of patients with nonfatal influenza infections also indicated that influenza virus spread was highly spatial, with significant variation in the degrees of inflammation and epithelial damage between bronchi within individual hosts [30]. Immunohistochemistry-based analysis of influenza-infected ferrets revealed that different subtypes of influenza virus all exhibited spatial viral spread, with notable differences in the spatial (and temporal) signatures of viral infection across the subtypes examined [15].

**Figure 1:**
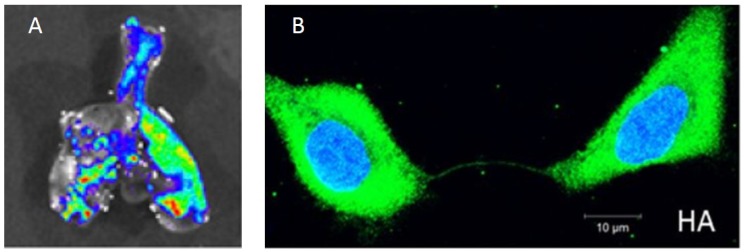
Experimental findings of within-host influenza virus spread. (**A**) Influenza virus spread visualized through bioluminescent imaging. The figure shows fluorescence from excised lungs of infected mice. Figure reproduced from [31]; (**B**) The genome and proteins of influenza virus can be transferred between cells via intercellular pathways called tunneling nanotubes. Figure reproduced from [34].

Recent advances in the development of recombinant viruses expressing fluorescent reporters in particular have greatly advanced our ability to understand the spatial aspects of within-host influenza dynamics. For example, Manicassamy et al. used a GFP-expressing recombinant virus to examine the localization and cellular tropism of the virus in mouse lungs [31]. Imaging of excised lungs four days post infection showed focal areas of viral infection (Figure 1A), similar to what was observed in human tissue samples. Fukuyama et al. engineered four distinct influenza viruses that stably encode different fluorescent reporter proteins [32]. By infecting mice with a mixture of these four ‘Color-flu’ viruses and tracking them independently, they observed clusters of the same fluorescent color within bronchial epithelial cells at 2 days post-infection. By day 5 post-infection, infected alveolar cells showed expression of single fluorescent proteins. Both of these observations point to highly localized (and possibly occasional long-distance) dispersal of virions. The authors also found that approximately 20% of bronchial epithelial cells were infected with more than one Color-flu virus at day 2 post-infection. This finding indicates that cellular coinfection during influenza infection is relatively common, consistent with a study in guinea pigs that found high IAV reassortant frequencies in the absence of segment mismatch [33]. The frequency of coinfection may have significant consequences for the spatial dynamics of infection (more below).

The development of viruses that stably express luciferase has allowed the visualization of longitudinal infection dynamics and spatial distribution within live hosts [35,36,37,38,39]. In mice, multiple studies have demonstrated the existence of clear viral foci in the lungs that spread in spatial extent before recovery from viral infection [35,36,37]. Subsequent studies have demonstrated the utility of these luciferase reporter viruses in tracking the spatiotemporal dynamics of infection in ferrets and in the context of pre-existing immunity [38,39], which is of particular interest given that partial immunity to influenza A viruses is widespread in human populations [40].

Altogether, there is an increasing body of experimental work in systems ranging from mice to humans that indicates that influenza infections are highly spatially structured. Along with this, recent studies are also providing a better understanding of the specific mechanisms that may be responsible for the establishment of this spatial structure. One clear factor is the spatial heterogeneity of cells with certain receptor distributions that mediate efficient viral attachment and therewith modulate cellular tropism. To elaborate, IAV primarily binds host cells through interactions with the galactose–sialic acid (SA) linkages present on the termini of complex glycan structures. The SA structures used by influenza viruses are structurally diverse, but are typically classified as either α2,3 or α2,6 based on the orientation of the bond between the galactose and sialic acid moieties [41]. The specificity of HA for α2,3 or α2,6 linkages is thought to be a key determinant of species and cellular tropism, with avian strains primarily binding α2,3 and human strains binding α2,6 [41]. Multiple studies have demonstrated a correlation between the presence of α2,6-linked SA receptors on the cell surface and virion binding and infection in human airway and nasal epithelial cultures, as well as within sections of human respiratory tissue [20,42,43,44,45]. Consistent with this, deep sequencing of different anatomical sub-compartments within the ferret respiratory tract revealed clear compartmentalization of viral variants based on the distribution of receptor structures [46]. Importantly, the ubiquity of SA receptors throughout the mammalian respiratory tract lumen may limit the spatial spread of virions. Release and efficient spread of newly produced virions depends upon the ability of the viral neuraminidase protein (NA) to efficiently cleave SA receptors from the surface of the infected cell [47,48]. In addition, the airway lumen contains an abundance of heavily sialylated host factors, such as pentraxins and mucins, that can bind virions and restrict their free diffusion [49,50]. Thus, both cellular and cell-free SA may act to restrict or structure the spatial spread of the virus by limiting free diffusion. This effect may be counteracted to varying degrees by the activity of the viral NA, which can differ between different influenza virus strains [51].

Other host factors beyond the distribution of SA receptors may also contribute to the spatial structuring of influenza virus populations during infection. The HA molecule requires activation via proteolytic cleavage in order to facilitate membrane fusion and subsequent infection. In humans, this cleavage is thought to be primarily mediated by the host proteases TMPRSS2 and HAT [52]. The expression of both TMPRSS2 and HAT is highly heterogeneous within the mammalian respiratory tract, owing to variation in expression levels between individual cell types [53,54]. Spatial heterogeneity in the availability of these pro-viral host factors may influence the spatial structuring of the virus. Further, the mammalian respiratory tract is not a static environment, and the directional flow of both air and mucus (via the mucociliary escalator) is likely to influence the spatial distribution patterns of virions [55], though experimental data on this are lacking.

Recent studies have suggested that influenza viruses may also be able to spread spatially via an entirely separate mechanism that does not depend on diffusion of extracellular virions. Specifically, Roberts et al. showed that viral proteins can spread between adjacent cells via intercellular actin pathways (“tunneling nanotubes” (TNTs)) without going through the standard budding and release process [56]. These proteins include the viral replicase machinery (nucleoprotein and polymerase proteins), as well as NS1. Subsequently, Kumar and colleagues showed that the genomes of influenza viruses can also spread between cells via these nanotubes (Figure 1B) [34].

Thus, influenza viruses may use at least two modes of transmission between cells: (1) the textbook process of extracellular spread by virions, and (2) the intercellular spread of viral genomes and proteins between neighboring cells. Infection therefore likely occurs at two characteristic spatial scales: a scale with the possibility of long-distance dispersal (with cell-free virions dispersing mostly locally but having a small possibility to infect cells at appreciable distances from the cells from which they budded) and a scale that involves highly localized spread (with intercellular pathways having the ability to form only between adjacent target cells). To our knowledge, the actual distances traveled by cell-free virions have not been measured experimentally, but given the spread of cell-free virions between hosts, it is likely that cell-free virions also have some degree of long-distance dispersal capabilities within a host. The relative importance of these two modes of spread may differ between infected hosts. For example, hosts with pre-existing anti-influenza immunity may clear cell-free virions more rapidly than naïve hosts, resulting in a more dominant role for intercellular viral spread in these individuals. Both of these processes also are subject to stochastic effects, which contribute to variation in the spatial distribution of IAV between infected individuals. For example, an infection may not successfully establish due to chance effects of where transmitted virus lands in the respiratory tract. Rare long-dispersal within-host dispersal events are particularly prone to stochastic effects, as was underscored by the study by Guarner et al. [29] which found that a variable fraction of lung tissue blocks were infected across fatally infected humans.

These two modes of viral spread can be considered bottom-up processes in that they impact the rate of viral spread via access to the resources necessary for replication. The spatial aspects of top-down processes that control viral spread, such as the activities of immune cells and cytokines in neutralizing virions, clearing infected cells, and rendering cells refractory to infection, have not been extensively studied to our knowledge. There is some evidence that inflammation of the respiratory tract can be spatially structured [57]. Questions that need to be addressed are thus how locally the immune system acts and the degree of spatial heterogeneity in the immune response across the respiratory tract. Further empirical work is needed to understand the potential for top-down regulation of viruses. However, because the respiratory tract is not a highly immune-privileged site, and numerous soluble components of the anti-viral immune response, such as antibodies and interferon, are thought to act at the tissue-wide or systemic level, we expect the spatial dynamics of influenza virus spread to be regulated more strongly by the bottom-up process of local target cell availability than by the top-down process of immune-mediated viral clearance.

In sum, the experimental findings we reviewed here indicate that human influenza viruses exhibit strong patterns of within-host spatial spread, driven predominantly by local movement of cell-free virions and the intercellular spread of viral genomes and proteins. In the next section, we review computational models that address the role that certain viral and host factors may play in shaping the spatial aspect of within-host influenza virus spread.

## 3. Computational Models of Spatial Within-Host Influenza Virus Spread

**Figure 2:**
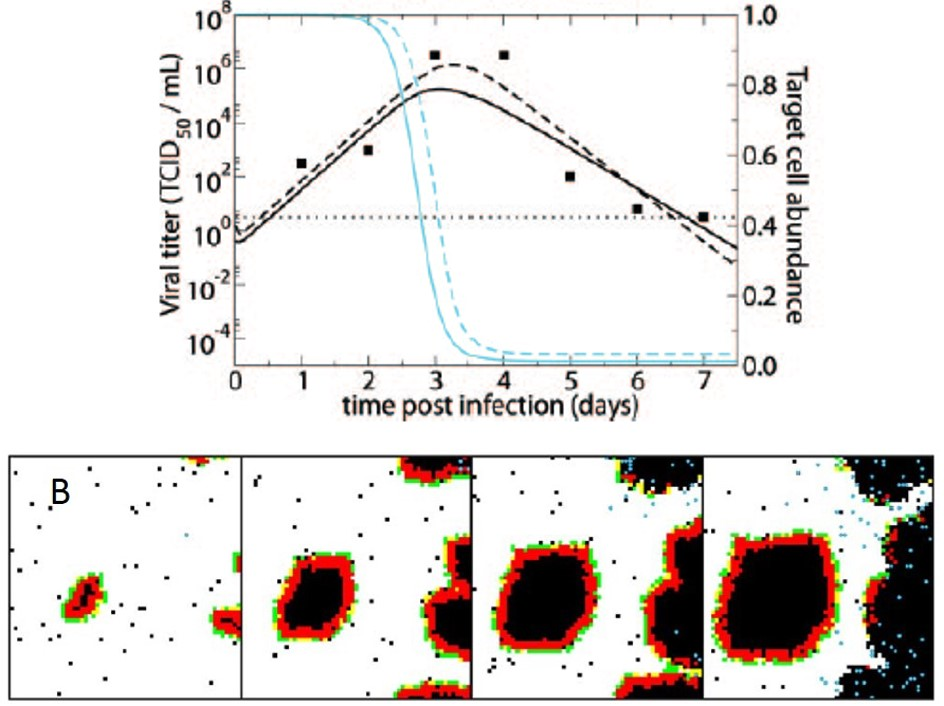
(**A**) Fit of a non-spatial, target-cell-limited within-host influenza model to viral load data from a human subject experimentally infected with influenza A subtype H1N1. The data are shown as black points. The black lines show model fits of viral load data. The blue lines show model-predicted declines in the number of target cells. Solid lines show the fits of the basic model; dashed lines show the fits of a more complex model with an eclipse phase before infected cells produce virus. Figure is reproduced from [60]; (**B**) Cellular automata model of within-host influenza infection under assumptions of local cell regeneration and localized recruitment of immune cells. Panels, from left to right, show the time evolution of the cellular automata model. Simulations reproduce the appearance of infected foci. Figure reproduced from [67].

The overwhelming majority of computational within-host influenza models do not incorporate a spatial aspect to viral spread. Despite this, they have provided insights into the processes regulating within-host virus dynamics [58,59]. These dynamics are typically characterized by exponential growth in viral load until approximately 2–3 days post-infection, with peak viral titers measured at approximately $10^{6}$ TCID50/mL [60]. Virus generally becomes undetectable within 5–6 days following infection [61]. The decline in viral load is often biphasic, with an initial rapid decline, followed by a longer, slower decline in viral load [60,62,63]. Figure 2A shows these characteristic viral load dynamics in a human subject experimentally infected with the H1N1 influenza A subtype. In some cases, the initial rapid decline is not observed, and the biphasic decline is characterized by a prolonged period of high viral loads, followed by a sharp decline in virus after several days [64].

Several non-spatial models have been able to reproduce these characteristic infection dynamics. The most basic versions of these models consider only target cells and virus, yet can reproduce the exponential growth of the viral population within a host, followed by an exponential viral decline once target cells have been depleted (Figure 2A) [60]. These models, however, fail to reproduce observed biphasic viral declines. More complex models have incorporated the host response, either phenomenologically through a time-varying infected cell clearance rate [64,65] or, more typically, by considering the role of interferon and cells of the innate and adaptive immune response [58,60,62,63,66]. These models have been able to reproduce many (and in some cases, all observed) patterns of viral growth and decline without unrealistic target cell depletion. The importance of the host immune response in regulating within-host influenza dynamics, identified by these non-spatial models, lay the groundwork for more complex models that explicitly account for spatial structure.

Incorporating spatial structure into within-host disease models can have important consequences. Most notably, parameter estimates inferred for spatial within-host models are often biologically more reasonable than those inferred for non-spatial models [68,69]. This has been shown for models of influenza viruses [68], as well for models of other pathogens [69]. This finding indicates that spatial aspects of viral spread might inappropriately bias inferred parameter values of non-spatial models. Spatial models also lead to qualitative predictions of viral dynamics that may be more biologically reasonable [26]. For example, under certain parameter regimes, one might expect a viral infection to become chronic or for viral load to equilibrate steadily; spatial models for chronic infections have been able to better reproduce these types of dynamics than non-spatial models, which have a greater tendency for viral infections to be stochastically cleared or to exhibit dampening oscillatory dynamics [26].

For influenza viruses, several spatially explicit within-host models have been developed in the last 10–15 years [23,70,71]. These models all allow virus to spread locally from infected cells to nearby susceptible cells. In some of these models, other processes, such as host cell regeneration and immune cell recruitment, are also spatially structured. The most common approach to explicitly model spatial aspects of within-host influenza virus spread has been through the implementation of agent-based models. These generally consist of a two-dimensional grid of cells and a defined set of rules that determine viral kinetics and cell death kinetics, among other kinetics, such as those of the host immune response. Beauchemin and colleagues showed that an agent-based model of this sort could successfully reproduce certain features of acute influenza infections, including the timing of peak viral load and the 5–7 day duration of infection [23]. However, in their simulations, the number of infected cells appears to grow linearly until viral load peaks; this stands in contrast to observed patterns of exponential viral growth over the first few days of influenza infection. More work needs to be done to determine whether and under what scenarios one would expect exponential viral growth in spatially structured influenza infections.

In a second study, Beauchemin considered the dynamical effect of factors occurring at different spatial scales [67]. Specifically, this study considered the regeneration dynamics of epithelial cells to occur either globally or locally. Which of these assumptions was adopted would clearly affect resource availability for the virus, and thereby shed light on the importance of this bottom-up process’s spatial scale in shaping the spatial distribution of the viral population. The study further considered the recruitment dynamics of immune cells to occur either at random or preferentially at infection sites. Considering these alternative assumptions allowed Beauchemin to evaluate the importance of spatial scale in the immune response’s top-down control of the viral population. Finally, this study considered different possible dispersal distances for the virus. Overall, the study found that local cell regeneration and short viral dispersal distances reproduced the observed empirical patterns, including foci of infected cells, better than other combinations (Figure 2B). Whether recruitment of immune cells was at random or localized at infected sites did not have an appreciable effect as long as cell regeneration was localized. Better experimental data are still needed to quantify cellular regeneration and to determine whether it should be expected to impact influenza virus dynamics over a 5–6 day period.

Following this work, Levin et al. assessed in more detail the importance of the host immune response in regulating spatial within-host influenza virus spread [72]. In this study, the authors showed that T-cells were unable to control the spread of influenza viruses with high replication rates. This inability of the host immune response to control the viral infection was due to delays in T-cell migration to the infection site. The results of this spatial model shed light on how the localized interaction between the immune system and the virus could result in the ability of some viral strains, but not others, to evade top-down control by the host immune response.

Despite the increase in their use, agent-based models are still computationally intensive and frequently do not allow for effective interfacing with data or analytical insight. Fortunately, several alternative approaches exist for modeling spatial aspects of within-host viral spread that do not rely on agent-based model simulations. One such approach is to compartmentalize the respiratory tract into several distinct ‘patches’, with low levels of viral transmission between one another. Within a patch, the virus is assumed to have equal access to all cells and is similarly targeted equally by all host immune responses. A study by Reperant et al. provides an example of this type of approach, in which viral dynamics are considered across three tissue compartments: the trachea/bronchi, the bronchioles, and the alveoli [73]. These compartments captured spatial heterogeneity in host cell types across tissues by differing in their initial number of susceptible target cells, in their viral clearance rates, and in their immunoglobulin distributions. By simulating this multi-patch compartmental model for a number of different influenza A subtypes, the authors found that these tissue differences lead to strain-specific variation in viral localization along the respiratory tract, and therewith differences in the onward transmission potential of different influenza subtypes. A second alternative approach makes use of partial differential equations (PDEs), which can deterministically simulate the dynamics of a viral population over both space and time. With PDEs, certain processes can occur locally while others can occur over more extensive spatial scales. For within-host influenza dynamics, for example, virus production, infection and death of cells, and immune activation can all occur locally, while viruses, cytokines, and certain cells can diffuse or migrate over more extensive ranges across space.

A third alternative approach to agent-based models is to consider space implicitly, rather than explicitly. This can be done by including saturating (instead of mass-action) terms in non-spatial mathematical within-host models [58]. While mass-action terms are often used in within-host models to describe virus infection of target cells, using a saturating term (such as a Michaelis–Menten term) to describe the infection process would allow for deviation from a well-mixed assumption. The rationale for using such a term is that when a virus is produced from infected cells, it cannot reach all target cells in a host. Instead, there are only a small number of target cells that are available to the virus to infect. Thus, the rate at which susceptible cells become infected can rapidly saturate even while many target cells remain susceptible. A final approach to modeling space implicitly is to allow for overdispersion of virus among target cells by assuming, for example, a negative binomial distribution for viral particles across host cells, rather than a Poisson distribution [74]. With overdispersion, a small number of target cells are infected with a large number of virions, while a large number of target cells might still be uninfected. Overdispersion can thus capture expected viral distribution patterns under the assumption of spatial viral spread.

While spatial within-host models have helped us understand how influenza virus infections spread within a host, the current literature has not addressed many open questions that seem particularly important in the context of spatial viral spread. One question is how the eclipse phase of infected cells impacts viral population growth and spatial spread dynamics. A second question is how cellular coinfection impacts the rate of viral spread. In a spatially structured infection, we expect substantially more cellular coinfection than in a non-spatial setting, where virus is spread more evenly over an entire population of cells. As such, the effect that cellular coinfection has on the rate of viral production will be critical to determining how quickly the viral population will spatially expand. Higher levels of cellular coinfection in a spatially structured setting will also impact viral reassortment rates [75], and thereby also impact the adaptive potential of influenza viruses. To our knowledge, these questions have yet to be addressed experimentally. Similar questions, however, have been considered elsewhere: in the ecological and evolutionary literature on range expansions. Indeed, this rich theoretical literature can be mined to inform us of answers to these questions, provided that we make effective analogies between processes identified in this literature and those acting on within-host viral populations.

## 4. Ecological Factors Driving Patterns of Spatial Spread

One of the most straightforward questions we can ask about within-host disease spread is how fast it will progress. On this question, the ecological literature has shown that spatially unstructured populations grow faster than their spatially structured counterparts when starting from small population sizes [76,77]. When population sizes are small, spatially unstructured populations are expected to grow exponentially, whereas spatially structured populations are expected to grow slower than exponentially (that is, sub-exponentially). This reduced growth rate is due to the lower relative availability of resources in spatially structured populations: resources are scarce in the centers of expanding populations, and resources are only abundant for those individuals at the very front of the expanding population wave. In the context of within-host viral spread, this means that infections that are highly spatially structured have constrained growth rates relative to those infections that are more spatially unstructured.

**Figure 3:**
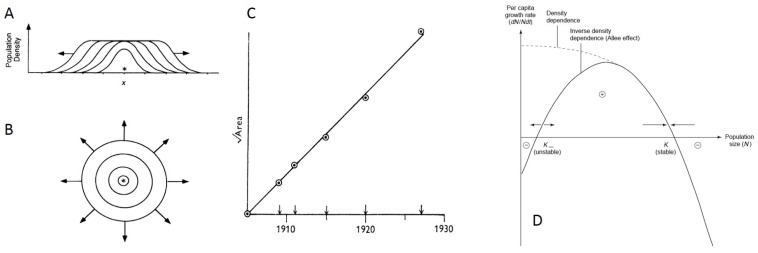
Patterns and dynamics of spatial spread from the ecological literature. (**A**) Populations expand as a traveling wave in a single spatial dimension; (**B**) Populations expand as a traveling wave in two-dimensional space. Figures (**A**) and (**B**) reproduced from [78]; (**C**) When populations expand in two spatial dimensions, the square root of the area that is inhabited is expected to grow linearly in time. Figure reproduced from [79]; (**D**) Types of density-dependence. Negative density-dependence (curve labeled “density dependence") occurs when per capita growth rates decrease with increases in local population densities. Allee effects occur when per capita growth rates (y-axis) first increase, and then decrease, with increases in local population densities or sizes (x-axis). Figure reproduced from [80].

While spatial structure is known to slow overall population growth, the ecological literature has also delved more specifically into what factors impact the rate of spatial population spread. In general, a population expanding outward from its point of origin is theoretically expected to spread as a “traveling wave”, that is, at a constant rate and with a wavefront shape that is maintained over time [78,79]. This traveling wave dynamic is expected when a population is expanding in a single dimension along a line (Figure 3A) or in two-dimensional space (Figure 3B), the latter of which would be more relevant to the within-host spread of influenza virus populations. If a population is expanding in a single dimension, the amount of occupied area is expected to grow linearly in time [79]. Alternatively, if a population is expanding in two dimensions, the square root of the occupied area is expected to grow linearly in time [79] (Figure 3C).

One important factor that affects the velocity of the traveling wave is the type of density-dependence that the population is subject to, where density-dependence generally refers to the relationship between local population density or size and individual (per capita) growth rates. Populations are said to undergo density-independent growth when an individual’s growth rate is not influenced by local population density. Negative density-dependence is said to occur when an individual’s growth rate decreases with increases in local population density, such that the maximum per capita growth rate occurs at small population sizes (Figure 3D). Positive density-dependence is said to occur when an individual’s growth rate increases with increases in local population density. Systems can be subject to multiple forms of density-dependence. For example, in populations with an Allee effect, there is a transition from positive to negative density-dependence with increases in local population density (Figure 3D). In populations that are strictly subject to negative density-dependence, the (asymptotic) velocity of the traveling wave is given by $\sqrt{4f^{\prime }(u)D}$, where *D* is the dispersal rate, measured in units of dispersal distance^2^/time, and $f^{\prime }(u)$ is the individual growth rate at low population density [78]. In populations with an Allee effect, the (asymptotic) velocity of the traveling wave is lower than that in similar populations without an Allee effect [81,82,83]. Thus, the type of density-dependence a population experiences will impact the velocity of its spatial spread.

While a spatially expanding population is generally expected to exhibit traveling wave dynamics, it may initially exhibit transient dynamics that differ from its asymptotic, long-term behavior. In many cases, these transient dynamics are expected to have a slower velocity than those of the asymptotic traveling wave [78,84,85], with the rate of spread expected to accelerate once the local population has reached a threshold density [86]. This expected increase in the rate of spatial population spread is an important theoretical finding that, if ignored, could lead to dramatic underestimation of the rate at which a population will ultimately spread and the total distance that it will ultimately travel. In the case of an Allee effect, some population expansions may even fail due to local populations failing to exceed certain threshold densities [83].

Unfortunately, little is known about density-dependence in viral populations specifically. Within a host, the characterization of density-dependence in a viral population would require determining how the number of viral progeny from a given intracellular viral particle depends on the multiplicity of infection of the cell the viral particle resides in. For some viral pathogens, host cell machinery may be the primary limiting factor. In this case, the population would be strictly subject to negative density-dependence. In influenza, there is some indication that an Allee effect may be at play. This expectation derives from a study that showed that over 90% of the time, singularly infected cells fail to produce viral progeny [87]. This failure to produce viral progeny stems from the failure of one or more of influenza’s eight gene segments to be delivered to the nucleus. The existence of these “semi-infectious particles” [88] that can produce viral progeny through complementation can therefore be thought of as bringing about positive density-dependence at low cellular multiplicities of infection (MOIs). With host cell machinery ultimately limiting viral production at high cellular MOIs, IAV growth may therefore be characterized by an Allee effect. As such, we may expect some infections to fail due to threshold population sizes not being reached, and we may expect the rate of viral spread to be slower for strains of IAV that have higher proportions of semi-infectious particles.

A second ecological factor that is known to impact the rate of spatial population spread is the frequency of long-distance dispersal events [89,90,91,92]. The primary ecological effect of long-distance dispersal events is an increase in the rate at which populations expand spatially [89,93]. This increase in the rate of spatial spread further leads to an overall increase in population growth rates because dispersed individuals have access to more resources than they would otherwise have had. In a study that compared two different modes of range expansion (exclusively short-range diffusion versus a combination of short-range diffusion and long-distance dispersal), populations were found to invade more quickly when long-distance dispersal occurred, even if these events occurred only rarely [91].

Given the importance of long-distance dispersal events on the dynamics of spatially structured populations, knowledge of how frequently virions disperse at these long distances within infected hosts appears critical. To the best of our knowledge, the frequencies of these events have not been quantified, either *in vivo* or *in vitro*. Clearly, transmission of influenza particles between hosts constitutes a long-distance dispersal event. While we know that influenza virions generally infect nearby cells, the extent to which virions travel long distances within a host is unknown. Intriguingly, the observed exponential growth of the viral population for the first 2–3 days following infection may be an indication that long-distance within-host dispersal occurs; in its absence, we would expect a pattern of sub-exponential viral growth. As mentioned above, long-distance dispersal mitigates, to some extent, the growth-slowing depletion of local resources (target cells, in the case of viruses), and thereby brings the rate of viral population growth closer to an exponential form. The frequency of long-distance viral dispersal in IAV infections should be investigated further, given the evidence in the ecological literature for the strong influence that dispersal rates have on rates of population spread.

Spatial heterogeneity is a third key factor that can impact the rate of spatial population spread. Spatially heterogeneous environments might be caused by irregularities in the landscape, such as unevenly distributed resources or barrier zones. While we normally expect populations to expand through space as a traveling wave, there is evidence that spatial heterogeneity can result in much more complex patterns. For example, Keeling and colleagues showed that heterogeneity across the landscape in terms of resource distribution and quality helped to explain why a disease outbreak traveled irregularly and was difficult to predict [93]. Another example can be found in a study by Sharov and Liebhold, who used empirical data and a spatially heterogeneous model to show that a single barrier zone could greatly reduce the rate of population spread [94]. Similar results have been found in other studies on the importance of barrier zones, which can serve to reduce the rate of spatial expansion or even halt it entirely [95].

These effects of spatial heterogeneity are an important consideration for within-host viral spread. We know that flu infections occur in a spatially heterogeneous environment. Across the length of the respiratory tract, the ‘landscape’ is heterogeneous in terms of cell densities and receptor structures. Progressing from the upper to the lower respiratory tract, we see an increase in the number of α2,3 SA binding receptors relative to α2,6 receptors, meaning there are fewer appropriate target cells for human influenza viruses to bind to [96,97]. This change in resource distribution could affect the pattern and rate of viral spread, helping to explain why many human influenza infections are confined to the upper respiratory tract. Furthermore, we can expect that within-host patterns of immune response would also add heterogeneity to the environment in the form of interferon diffusing as it is released from infected cells and immune cells moving through the system. This is an active area of study, and recent advances in within-host imaging techniques will no doubt greatly advance our understanding of within-host spatial heterogeneity and its effects on viral spread.

Finally, the presence of other species can strongly affect the ability of a species to invade. Competitors can act to reduce the availability of resources or alter the environment in other ways that make it more difficult for a species to disperse and survive. Unsurprisingly, most models suggest that the presence of a competitor will act to slow down the rate of spatial spread [98]. The competitor can still have this effect even if it is less fit than the focal species. This is especially true if the competitor is already established in the new location before the focal species arrives [92], but this is not a requirement. Similarly, predators can also slow down the rate at which a species can invade a new territory, and, depending on their distribution in the landscape and their time of release, they may even make it such that the invasion dynamics of the prey species can no longer be characterized by a traveling wave [78]. In the context of influenza, while the virus may not explicitly be subject to interspecific interactions, there are many parallels between host–virus interactions and classical ecological species interactions. For example, we can consider components of the immune response to be either competitors or predators acting on the virus. In particular, exposure to interferon-α is known to make cells refractory to viral infection, thereby reducing the number of susceptible target cells available to a virus. Interferon-α could therefore potentially be considered an asymmetrical competitor of IAV within a host. The depletion of susceptible target cells by interferon-α would act to slow down the rate of viral spread within a host. The cellular and humoral immune responses of hosts could instead be thought of as predators of the within-host IAV population by neutralizing free virus or killing infected cells. Viruses infecting hosts with pre-existing immunity would thereby experience top-down, predator-like control from the immune system. This dynamic would lead to a slower rate of viral spatial spread, and potentially the abrogation of a traveling wave form.

In sum, our understanding of ecological dynamics can help us to better understand within-host viral dynamics and to fill in some of the gaps in our knowledge about factors that may impact rates of viral spread. To consider how these spatial aspects of viral spread will in turn impact the genetic structure of the viral population, we next turn to the evolutionary literature.

## 5. The Consequences of Spatial Spread on Population Evolution

The evolutionary literature provides insight into how spatial spread impacts patterns of population genetic diversity, how it impacts the processes of purifying and positive selection, and how spatially distinct selection pressures may shape population phenotypes. Here, given the intrinsically spatial aspect of influenza virus spread within hosts, we review this literature and again make ties to observations from the flu field where possible.

**Figure 4:**
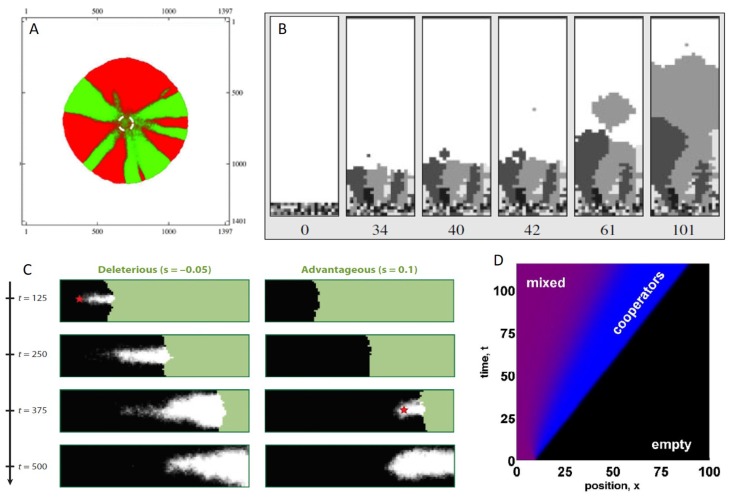
The effects of spatial spread on a population’s evolutionary dynamics. (**A**) Local movement in two-dimensional space leads to the generation of genetically homogeneous ‘sectors’. Figure reproduced from [109]; (**B**) Intermediate levels of long-distance dispersal result in major reductions in genetic diversity, as described by the ‘embolism effect’. Panels, from left to right, show time evolution of the simulation. Figure reproduced from [105]; (**C**) Mutations can ‘surf’ to high frequencies, regardless of whether they are deleterious (left), beneficial (right), or neutral (not shown). Figure reproduced from [100]; (**D**) Spatially expanding populations can select for cooperative phenotypes at the leading edge. Figure reproduced from [110].

An important effect of spatial population expansion is a significant reduction in population genetic diversity. This effect is one of the more robust effects of spatial spread, with a large number of studies showing that genetic diversity is rapidly eroded when population expansion occurs locally, as with a range expansion [99,100,101,102,103]. In the case of spatial expansion in two dimensions, this reduction in genetic diversity from stochastic founder effects results in sectors that are genetically homogeneous [101] (Figure 4A). Intriguingly, these patterns are consistent with a recent analysis of within-host viral populations in individuals experiencing acute influenza infections [104]. Specifically, McCrone and colleagues found that stochastic effects dominated in the structuring of within-host flu populations, and that, despite high viral titers, only 57% of single nucleotide variants from early samples were still present in later samples from the same individual when samples were taken one or more days apart. These results are consistent with the phenomenon of spatial spread, where rapid drops in standing genetic variation would be expected further into the range expansion due to genetic drift at the wavefront. Rapid losses of genetic diversity were also evident in a mouse model for influenza infection, where the authors found, using four distinct colors of fluorescently labeled viral proteins, that the majority of individual alveoli only showed the presence of a single color [32]. This indicates that at the furthest extent of within-host viral spread, spatial founder effects and bottlenecks appear to be at play.

Several factors have been identified in the population genetic literature that will modulate the extent to which the genetic diversity of a spatially expanding population will be eroded. In most cases, these factors have clear analogs for within-host viral populations. First, the ‘dispersal kernel’ is known to affect the rate at which populations will lose genetic diversity, where the dispersal kernel quantifies the distribution of distances individuals in a population will seed their progeny. Intuitively, one might think that higher levels of long-distance dispersal will always mitigate the loss of genetic diversity. However, Bialozyt and colleagues showed instead that increases in the number of long-distance dispersal events will counterintuitively first have the effect of reducing genetic diversity [105]. Further increases in the number of long-distance dispersal events will then act to increase levels of genetic diversity again. This pattern results in the minimum level of population genetic diversity being present at some level of long-distance dispersal. This pattern results from what has been termed an ‘embolism’ effect (Figure 4B), where rare long-distance dispersal events lead to single individual founders with substantial replication resources surrounding them. The rapid expansion of these single individual founders leads to dramatic reductions in the overall population’s genetic diversity. The dispersal kernel, as one might expect, will also impact the genetic ‘patchiness’ of the population across space [106]. In light of the two possible modes by which flu viruses infect target cells, the relative roles of cell entry through receptor binding by free virus versus cell entry through tunneling nanotubes will likely be important in understanding patterns of genetic diversity in within-host flu populations. If TNTs are a major source of cellular infection, as they may be in previously infected individuals with strong antibody responses, then dispersal is expected to be more highly localized, and long-distance dispersal events will be fewer. However, whether this will lead to higher or lower levels of genetic diversity relative to a case with higher levels of cell entry via receptor binding by free virus is unclear, given that the relationship between genetic diversity and the number of long-distance dispersal events is non-monotonic [105].

A second factor affecting the rate at which population genetic diversity will be lost in a spatially expanding population are the life history characteristics of the population. For example, it has been shown that a juvenile, non-reproductive stage in a life cycle reduces the rate of genetic diversity loss in populations [107]. This is because a juvenile stage slows down the colonization process and allows for more genetic diversity to accumulate at the wavefront. An ‘eclipse’ phase in viral populations is analogous to this juvenile stage: infected cells are not productive immediately following infection; rather, it can take several hours for viral progeny to be produced. For influenza, the duration of this eclipse phase has been quantified experimentally, with most recent estimates being on the order of 2–4 h [108].

A third factor affecting the extent to which genetic diversity will be eroded is how an individual’s reproductive rate depends on nearby population density. For example, theoretical studies have shown that Allee effects have the potential to maintain genetic diversity in a spatially expanding system [101,102]. A higher level of genetic diversity is maintained in populations with an Allee effect because, in these cases, it is not only the furthest members of a population that contribute to the expanding population. As discussed in the previous section, Allee effects may be at play in within-host viral populations that require complementation, including influenza viruses.

A fourth factor affecting levels of genetic diversity in spatially expanding systems is the extent of spatial heterogeneity. Specifically, Wegmann and coauthors showed that environmental heterogeneity leads to loss of genetic variation within similar regions and further leads to greater genetic differences between regions [111]. This finding may be applicable to within-host viral populations that exist across regions with different cell types. For example, within-host regions of cells having predominantly α2,3 versus α2,6 sialic acid receptors might result is less genetic variation within each region and greater levels of population genetic differentiation between regions. In fact, this is exactly what was observed when Lakdawala and colleagues examined the distribution of viral sequence variants between tissue compartments within ferrets that differ in receptor distribution [46].

Since evolutionary theory provides clear predictions as to how these four factors should influence patterns of genetic diversity in spatially structured populations, the opportunity is ripe for these predictions to be tested empirically in viral systems. By using barcoded viruses and manipulating these factors in an independent and highly controlled fashion, one would be able to assess whether viral populations are indeed governed by the processes identified as important in the evolutionary literature. Barcoded viruses have already been used to measure losses of genetic diversity during transmission events between donors and recipients [112], and thus could in principle be applied in a similar fashion to examine patterns of genetic diversity loss *in vitro* or *in vivo*.

Beyond impacts on population-level patterns of genetic diversity, populations that are spatially expanding are known to be subjected to a phenomenon called “surfing” [100,101,113,114], whereby genetic variants present on the wavefront of an expanding population may rapidly rise to high frequencies due to the dominance of genetic drift in the small wavefront populations. With the process of genetic drift (over selection) dominating at the wavefront of an expanding population, de novo mutations (whether beneficial, deleterious, or neutral) that occur at the right place at the right time can rise to high frequencies and even fix in populations (Figure 4C). Since, in many systems, the majority of mutations appear to be deleterious, this surfing phenomenon results in deleterious mutations fixing at considerably higher rates in spatially expanding populations than in populations that are growing in the absence of a spatial dimension [100,115]. As such, these spatially extended systems are expected to carry an “expansion load” [116,117], defined as the deleterious mutation load a population carries that is due to spatial founder effects from small populations at the wavefront. While there is no evidence yet for within-host viral populations being subject to the surfing phenomenon and to expansion loads, one should theoretically expect this to be the case. This is because most RNA virus mutations are known to be deleterious, with recent experimental findings providing evidence for this specifically for influenza virus [118].

While this surfing phenomenon is also relevant to beneficial mutations, the consequences of genetic drift dominating at the wavefront results in lower rates of beneficial mutation accumulation in a spatially expanding population than would be anticipated in population expanding in the absence of a spatial dimension. This is for two reasons: first, beneficial mutations are rare, so, relative to deleterious mutations, de novo mutations are unlikely to be beneficial. Second, if a beneficial mutation does arrive in the right place at the right time, it is unlikely for it to be brought to high frequencies through selection because of the dominance of genetic drift at the wavefront. This surfing phenomenon is thus known to slow the rate of adaptation of spatially expanding populations, and could even lead to fixation of deleterious mutations within hosts. Spatial within-host dynamics may therefore provide a mechanism to explain why RNA viruses, including influenza, appear to carry deleterious mutation loads [119,120,121].

Finally, spatially expanding populations may select for different phenotypes than ones that do not have a spatial dimension. This would occur, for example, if individuals residing at the wavefront experience different selection pressures from the ones residing at the interior of the population. Rather than this being an unlikely case, different selection pressures at different points in the population range are theoretically expected in many situations. At the wavefront, resources are relatively abundant, and individuals with a high intrinsic growth rate (“*r*”) are known to outcompete others. In contrast, in the interior of a population range, resources are limiting, and individuals with more efficient resource use do best (i.e., those individuals with higher basic reproduction numbers, $R_{0}$). This difference in *r* versus $R_{0}$ selection pressures has been considered in the context of infectious diseases, and is at the core of why epidemic pathogens (with abundant host resources) are expected to evolve to higher virulence compared to endemic pathogens (with scarce resources) [122]. Analogously, one would expect more virulent viruses to be selected for at the wavefront of an expanding population, compared to the interior [76], regardless of whether we are considering the viral population to be expanding within hosts or across the globe. In the context of within-host flu dynamics, spatial spread may therefore select for phenotypes that kill infected cells more rapidly but have higher rates of viral production. Another within-host phenotype that may be at least partly under viral genetic control is the viral dispersal kernel. Given theoretical findings that the evolution of long-distance dispersal is favored during an expansion process [123], perhaps one might even expect influenza virus to evolve a preference for cell entry via budding over cell entry via TNTs. Finally, a recent study intriguingly found that cooperative phenotypes have a selective advantage along the wavefront of expanding populations [110] (Figure 4D). This theoretical finding is particularly relevant to recent work examining the evolution of viral cooperation, collective interactions, and more generally, the budding research area of “sociovirology” [124].

In sum, the spatial aspect of within-host viral spread will generally reduce viral genetic diversity, slow the rate of viral adaptation, more easily enable the fixation of deleterious mutations, and result in the evolution of viral phenotypes that may be advantageous for only a subset of the viral population.

## 6. Discussion

We have reviewed the current understanding and open questions regarding patterns and mechanisms of within-host viral spread from both empirical and computational perspectives. Our ability to visualize within-host spatial structure has improved greatly thanks to advances in imaging techniques, particularly the use of fluorescent reporters and luciferase-expressing viruses. The recently discovered ability of viruses to spread directly from cell to cell via ‘tunneling nanotubes’ is an exciting development, but the feasibility and frequency of long-range dispersal remain unknown. Spatially explicit and spatially unstructured models have both been developed to understand viral kinetics within hosts. Spatially unstructured models are far more common, and increasingly have been effective at quantitatively recovering observed patterns of viral load kinetics and immune cell kinetics. However, ignoring spatial structure in the infection processes can lead to biased or incorrect estimates of parameter values and to qualitatively different behavior from that expected from a spatial process. Compared to spatially unstructured models, spatially structured models are potentially more informative for understanding the roles that cellular multiplicity of infection and long-distance dispersal play in shaping viral dynamics. Spatially structured models are also likely to be more informative for understanding evolutionary changes in within-host viral populations, as spatial spread is known to affect patterns of genetic diversity and fixation probabilities of both beneficial and deleterious mutations. Further, compared to spatially unstructured models, spatial models have the ability to more effectively incorporate spatial heterogeneity and thereby ascertain the consequences of this heterogeneity on viral dynamics and evolution

Due to the importance of the spatial aspect of within-host viral spread, we turned to the ecological and evolutionary literature to provide theoretical insight into the population dynamics and genetics of spatial within-host viral spread. Ecological and evolutionary studies indicate that the within-host spread of a virus should be strongly influenced by its own dispersal patterns and life history characteristics, the type of density dependence it is subject to, the types of interactions it has with the immune response, and the spatial heterogeneity in the host environment. The ecology literature has also given us insight into critical gaps in our knowledge about within-host viral spread. Specifically, we need more empirical data on viral density-dependence and the extent to which Allee effects are present in the system. Studies to determine the distance that viruses disperse would also greatly improve our understanding of what regulates the rate of within-host spread. Long-range dispersal greatly increases the speed of invasion, even if that dispersal is rare, but the extent to which long-range dispersal occurs in influenza viruses is currently unknown. The evolutionary literature has provided us with theoretical expectations for how the genetic structure of a viral population will change over time in an influenza virus infection, and the effect that spatial spread may have on the ability of a viral population to adapt. These predictions should be tested empirically, using available imaging and sequencing approaches.

Perhaps most importantly, the ecology and evolution literature has the potential to inform the development of control strategies. Current control strategies focus on treatment and prevention of infection using drug therapies and vaccination. These interventions introduce antibodies or antivirals into the system, both of which are functionally similar to predators from the standpoint of a virus in a host. They can be very effective under the right circumstances, but vaccines are notoriously difficult to formulate due to the rapid evolution of seasonal influenza strains, and antiviral resistance is not uncommon. In order to control an infection, the host must be able to contain the virus and prevent its ongoing spread. In vivo, this seems to be possible because the immune system responds quickly to the location of infection, and the virus ultimately runs out of local susceptible cells to infect. Early intervention is likely to be most effective, not only because there are fewer total virions and infected cells, but because influenza may be subject to strong Allee effects. Control efforts should be focused on reducing the maximum intrinsic growth rate of a population, which is not necessarily the intrinsic growth rate of a population when population density is low [84].

Studies of spatial heterogeneity suggest that introducing a barrier zone can be a very effective control strategy [93]. In wildlife populations, an artificial barrier has been successfully introduced at times to prevent the spread of rabies by depositing vaccine-laden food items [125]. While it is likely not possible to introduce a physical barrier within the host’s respiratory tract, the barrier zone concept is somewhat analogous to the potential for local action by the immune system to protect susceptible cells surrounding the site of infection, thus restricting the spatial spread of virus. For example, this kind of spatially structured immune response has been observed on the skin of mice infected with vaccinia virus [126,127].

Finally, influenza infection could potentially be controlled by introducing defective interfering particles (DIPs) into the system. DIPs are naturally occurring during infections, and they essentially parasitize wild-type virus, reducing the amount of infectious offspring that is produced from cells coinfected with DIPs and wild-type virus. The ability of DIPs to interfere with wild-type virus depends on the local cellular MOI, because in the absence of coinfection with a wild-type (“helper”) virus, DIPs cannot replicate [128]. Studies in both mice and ferrets have shown that DIPs can modify within-host influenza virus dynamics, decreasing peak viral loads and delaying its timing [129]. Further, the administration of DIPs can reduce influenza symptoms and virulence [129]. As reviewed in [130], the majority of models that incorporate DIPs have focused on *in vitro* viral dynamics due to the importance of controlling DIP accumulation when passaging stock viruses and producing vaccines [131,132]. However, Laske et al. [133] developed an intracellular model of replication dynamics, the results of which support the hypothesis that DIPs may be able to outcompete wild-type virus because they are shorter and can be replicated faster. Additionally, Farell et al. [134] use a within-host model that incorporates implicit spatial structure to show that DIPs can reduce peak viral load if administered early enough in the course of infection.

We have focused this review on influenza A viruses, which are a well-studied group of viruses that exhibit strongly spatially structured infections when infecting mammalian hosts. It is possible, and quite likely, that specific patterns of spatial spread differ, at least to some extent, between different IAV subtypes (e.g., H3N2 and H1N1, both circulating in humans). Differences in the spatial dynamics between IAV subtypes (and types) may arise due to differences in viral replication rates, the extent to which these viruses produce semi-infectious and defective particles, and the extent to which these viruses infect previously infected hosts. Indeed, because influenza A viruses undergo antigenic drift, most infected individuals are not naïve but have some degree of immunity. In addition to differences in the dynamics of spatial within-host spread that are due to differences between IAV subtypes, differences in these dynamics are likely to be present across hosts due to differences in their infection histories.

While we have focused here on influenza viruses, insight from the ecological and evolutionary literature is also applicable to a broad range of other viral infections. Accounting for the ecological and evolutionary dynamics of within-host spatial spread will deepen our understanding of the behavior and outcomes of a wide variety of viral infections and potentially lead to new conceptual advances in infection control strategies.
